# Insulin resistance in the retina: possible implications for certain ocular diseases

**DOI:** 10.3389/fendo.2024.1415521

**Published:** 2024-06-17

**Authors:** Zhaoxia Zheng, Xiaobing Yu

**Affiliations:** ^1^ Department of Ophthalmology, Beijing Hospital, National Center of Gerontology, Institute of Geriatric Medicine, Chinese Academy of Medical Sciences, Beijing, China; ^2^ Graduate School of Peking Union Medical College, Beijing, China

**Keywords:** insulin resistance, retina, diabetic retinopathy, glaucoma, age related macular degeneration

## Abstract

Insulin resistance (IR) is becoming a worldwide medical and public health challenge as an increasing prevalence of obesity and metabolic disorders. Accumulated evidence has demonstrated a strong relationship between IR and a higher incidence of several dramatically vision-threatening retinal diseases, including diabetic retinopathy, age-related macular degeneration, and glaucoma. In this review, we provide a schematic overview of the associations between IR and certain ocular diseases and further explore the possible mechanisms. Although the exact causes explaining these associations have not been fully elucidated, underlying mechanisms of oxidative stress, chronic low-grade inflammation, endothelial dysfunction and vasoconstriction, and neurodegenerative impairments may be involved. Given that IR is a modifiable risk factor, it may be important to identify patients at a high IR level with prompt treatment, which may decrease the risk of developing certain ocular diseases. Additionally, improving IR through the activation of insulin signaling pathways could become a potential therapeutic target.

## 1 Introduction

An acceleration of the prevalence of insulin resistance (IR) and metabolic syndrome (MetS) is becoming a worldwide public health challenge with an increasing rate of obesity (1). Evidence has indicated that IR has a strong association with the development of the cardiovascular diseases (2, 3). Insulin resistance is a pathological condition in which the ability of insulin to influence glucose uptake via insulin-dependent transportation is impaired, thus a higher-than-normal concentration is required to maintain a normal glucose level (4). That is, insulin-dependent cells fail to respond to a normal circulatory level of insulin due to decreased insulin sensitivity. IR is the hallmark of type 2 diabetes mellitus (T2DM) and MetS, representing a common feature of a cluster of metabolic abnormalities in T2DM and MetS, such as obesity, hyperinsulinemia, dyslipidemia, and high blood pressure (5).

In detail, increased IR, conversely correlated with insulin sensitivity, could be attributed to reduced capacity of insulin receptors to bind with insulin. At the molecular level, the explanations for decreased affinity of insulin receptors may include a declining number of insulin receptors, a mutation in insulin receptors, and autoimmune antibodies against insulin receptors (6). The interaction of insulin receptors with their ligands has been considered a key determinant for functional signal transduction, any interference in this process would lead to IR (6). Previous studies mainly investigated the effect of IR on traditionally thought insulin-sensitive tissues, such as liver, skeletal muscle, and adipose tissue, but accumulating evidence has suggested that IR plays a crucial role in the central nervous system (CNS) and retina (7, 8), since insulin signal pathway is a key determinant for cell survival, especially for neural cells (9). Although inconsistent results exist regarding the connection between IR and ocular diseases, increasing studies have postulated that IR is associated with glaucoma and several retinal diseases, particularly diabetic retinopathy (DR) and age-related macular degeneration (AMD), which account for the majority of vision loss in middle-aged and elderly populations (10, 11). To the best of our knowledge, currently established treatment options for retinal diseases are mainly limited to advanced stages, which only partially suffice to slow disease progression. Consequently, identification and treatment of IR in retina, which acts as a potential common pathogenesis and a modifiable risk factor (12), are imperative for the early management of these retinal diseases. In this review, we tend to summarize current evidence supporting the association between IR and certain ocular diseases, including DR, AMD, and glaucoma, and further explore the underlying pathophysiological mechanisms explaining these associations. Given that IR is a modifiable risk factor, investigating the intrinsic relationships between retinal diseases and IR would provide new insights for the administration of certain ocular diseases and even become potential therapeutic targets.

## 2 Insulin and insulin receptors in the retina

Insulin signal pathways are triggered by the binding of insulin to insulin receptor, a heterotetramer composed of two extracellular α subunits and two transcellular β subunits linked by disulfide bonds. Binding of insulin to the α subunit of insulin receptor leads to structural changes in the β subunit and triggers its intrinsic tyrosine kinase activity (13). The insulin receptor autophosphorylates its β subunit in tyrosine residues and initiates a cascade of downstream effects, such as recruitment of protein substrates of the insulin receptor kinase including the insulin receptor substrates (IRSs), SHC transforming protein, and casitas B-lineage lymphoma (Cbl) (13, 14). IRSs are key substrates in insulin signal transduction by binding to phosphatidylinositol-3-kinase (PI3K) and inducing downstream pathways. Indeed, any factors that reduce IRS-1 phosphorylation or induce serine IRS-1 phosphorylation at the 307 site by some kinases, such as IKKβ/NF-κB and c-Jun N-terminal kinase (JNK), would impair insulin signal transduction and lead to insulin resistance (15, 16). In addition to common target organs (liver, skeletal muscles, adipose tissue, etc.), the insulin receptor has been reported to be expressed constitutively and broadly throughout the retina on neuronal, endothelial, and retinal pigmented epithelial (RPE) cells (17). However, different to the fluctuation with circulating insulin levels during periods of fasting and feeding in the liver and skeletal muscle, the activity of insulin receptor in retina is maintained in a relatively stable state owing to the exquisite regulation of blood retina barrier (BRB) between the plasma and neural retina (18). And the transport of insulin across the BRB to retinal endothelial cells is significantly slower than other vascular beds. Collectively, the retina is not a major target for immediate nutrient metabolism as is skeletal muscle, but the steady-state transport of insulin to the neural retina may provide a stable trophic signal (18). In addition, another interesting observation is that retina and numerous other extra-pancreatic tissues express insulin and insulin-like molecules as a paracrine hormone (19).

**Figure 1:**
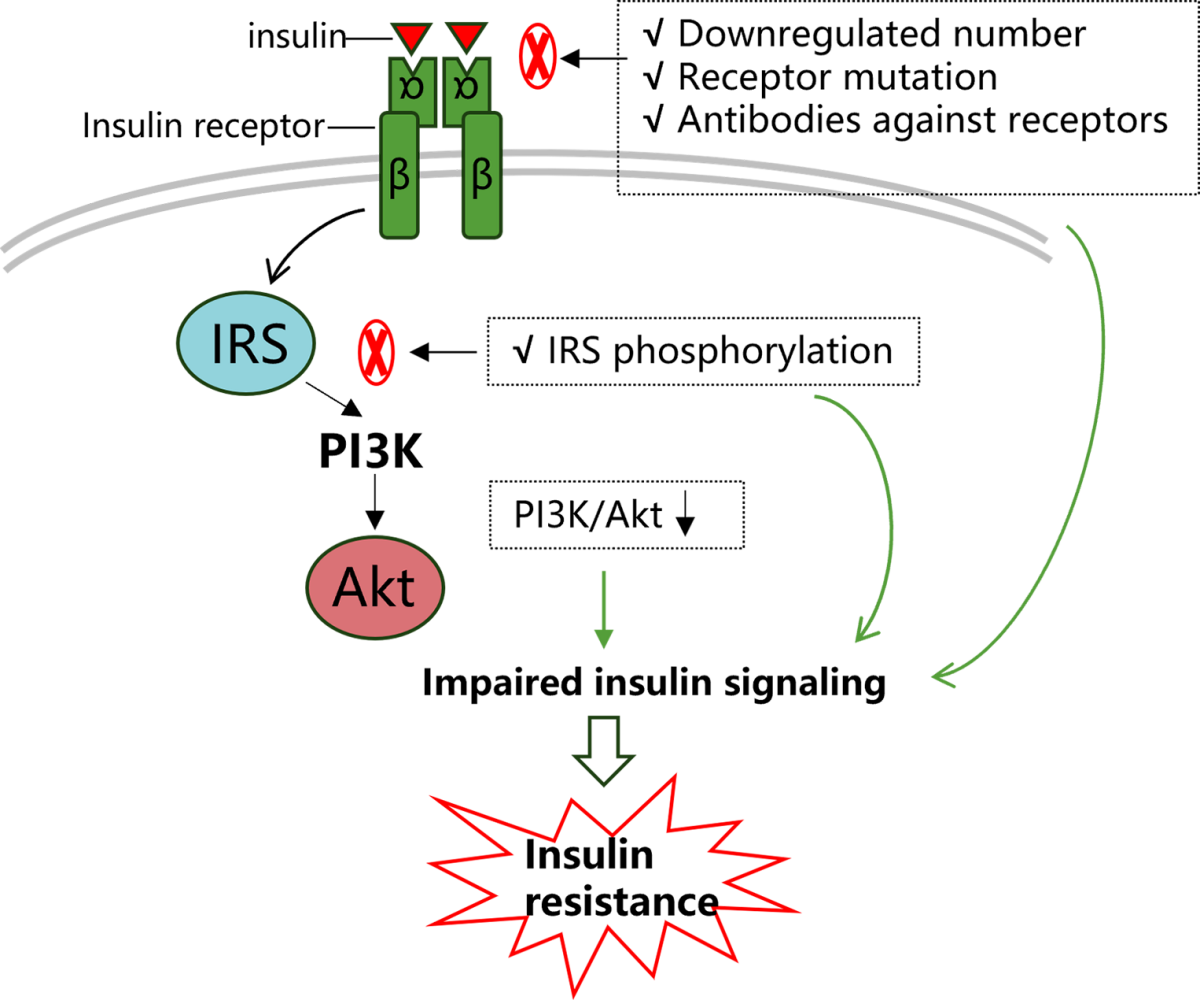
Insulin signaling transduction and insulin resistance. Insulin signaling pathways are complicated, among which the IRS/PI3K/Akt pathway has received much attention as it is well conserved and plays a role in many cellular processes. Firstly, as the capacity of insulin receptors to bind with ligands is a key factor in transduction, a downregulated number of insulin receptors and decreased insulin receptor affinity would significantly restrict normal insulin signaling. The latter could be due to mutations in insulin receptors and autoimmune antibodies against insulin receptors. Subsequently, any factors that reduce IRS phosphorylation or induce IRS phosphorylation at certain sites (such as serine IRS-1 phosphorylation at the 307 site) would impair downstream pathway transduction. Collectively, in this process, any abnormalities in these agents may impair normal insulin signaling transduction and lead to insulin resistance.

In general, regardless of insulin action or production site, insulin signal characteristics in retina resembles what has been described in other system. Specially, the insulin receptor/IRS/PI3K/Akt pathway has received much attention and plays a role in many cellular processes, such as glucose uptake and cellular survival (20) (**Figure 1**). Meanwhile, insulin resistance associated with diabetes and obesity appears to be due to pathway-selective impairment of PI3K/Akt signaling, whereas SHC/Ras/mitogen activated protein kinase (MAPK) is largely unaffected, thereby tipping the balance of insulin’s actions (21). This imbalance would favor abnormal vasoreactivity, mitogenesis, and other pathways implicated in microangiopathy.

## 3 The measurement and quantification of IR

Methods to quantify IR could be divided into two categories: dynamic tests and static tests. Dynamic tests mainly encompass the hyperinsulinemic euglycemic glucose clamp (HEGC), the frequently sampled intravenous glucose tolerance test (FSIVGTT), and the oral glucose tolerance test (OGTT) (22). Among these dynamic tests, the HEGC technique is the gold standard for measuring IR, and OGTT is the most applied dynamic test. Nevertheless, dynamic methods are resource and time consuming, thus limiting their application in clinical practice. On the contrary, static tests to estimate IR are more available and simpler, among which the homeostatic model assessment of IR (HOMA-IR) is the most commonly used, which is calculated just with the parameters of fasting glucose and insulin (23). Meanwhile, various glycemic indies and serum biomarkers, including insulin, C-peptide, glucose levels, and glycated hemoglobin (HbA1c), are measured to assess pancreatic β-cell function and sensitivity. Other anthropometric characteristics are also used to evaluate IR, such as body mass index (BMI), waist-to-hip circumference ratio (24).

## 4 The presence and evidence of IR in the retina

### 4.1 Diabetic retinopathy

T2DM and MetS are two representative diseases of IR, and the latter is considered preclinical diabetes. Individuals with MetS were reported to have a 5-fold increased risk for the development of T2DM (25). Although the diagnosis of T2DM is definite with blood glucose values meeting specific requirements, many patients may experience preclinical diabetes. In this prediabetes period, patients would undergo an unstable hyperglycemic state with a series of metabolic disorders, also known as MetS (4). IR is thought to be involved in the pathophysiology of MetS and diabetes. That is, T2DM is characterized in the first step by the fact that insulin action is impaired and pancreatic β-cells need to synthesize and secrete more insulin to compensate for this IR state, which could lead to hyperinsulinemia. In later stages, the insulin production is lower than normal, and hypoinsulinemia would be observed with the progressive reduced number and functional exhaustion of pancreatic β-cells (26).

DR, one of the most important microvascular complications in diabetes, has been regarded as a neurovascular coupling impairment recently, which includes both retinal neurodegeneration and vasculopathy (27). Whereas hyperglycemia has long been thought to be the primary driver in the progression of diabetic complications, impaired insulin signaling is also suspected to contribute to the retinal pathology. An experimental study suggested that both restoration of glycemic control and retinal insulin signaling can normalize diabetes-induced retinal abnormality via mediating Akt kinase activity, the expression of inflammatory mediators, and lipid synthetic pathway (28).

Meanwhile, it is easy to understand that intensive hypoglycemic therapy in T1DM has been shown to effectively prevent the occurrence and progression of DR (29). However, previous trials evaluating the effects of intensive glycemic control in patients with T2DM have provided inconsistent results (30, 31). The difference of intensive glycemic control may imply that beyond hyperglycemia, inherent risk factor of IR could contribute to the microvascular outcomes in the cohort of T2DM. Similarly, another evidence for the presence of IR has been shown in a study investigating structural and functional changes in adolescents with type 1 and type 2 diabetes. Adolescents with T2DM appeared to be more affected than those with T1DM with more significant multifocal electroretinography (mfERG) implicit time delays and retinal thinning (32). The authors attributed the reason for presenting with more abnormalities in T2DM to the explanation that T2DM patients are faced with more challenges that are inherent to IR, such as elevated BMI, hyperinsulinemia, and dyslipidemia. Additionally, Karaca et al. found that patients of MetS had similar neurodegeneration pattern to those with diabetes with no presence of DR (4). This indicates that inherent mechanisms of MetS, such as IR and adipose tissue-derived inflammation, could contribute to neurodegeneration independent of the diabetic level of hyperglycemia (4).

In addition to reflecting the role of IR in DR indirectly, recent studies have also quantitatively evaluated the relevance of IR-related parameters and DR. It is suggested that IR exacerbates DR, even as an independent predictor (33–35). Moreover, recent advancements in optical coherence tomography (OCT) technology have offered a non-invasive, repeatable monitoring tool for DR with its automated segmentation function. In a previous study, the insulin secretory/resistance parameters, including the fasting insulin level and HOMA-IR and HOMA-B scores in T2DM without DR, revealed the very early microvascular changes measured by OCT angiography (24). Likewise, our previous study indicated that IR (reflected by HOMA-IR values) was an independent risk factor for retinal ganglion cell-inner plexiform layer (GC-IPL) thinning, reflecting retinal neurodegeneration in early T2DM (36). Additionally, a relationship between cystoid macular edema and IR was found by Zapara et al., as IR is a proinflammatory state (37).

Previous experimental studies at the cellular level can also help shed light on the role of IR in DR. Müller cells, activated in the initial stage of DR with increased expression of glial fibrillary acidic protein (GFAP) and other cytokines, play an important role in maintaining retinal homeostasis (38). Experimental data have demonstrated that similar with that in retinal endothelial cells, hyperglycemia environment incudes increased tumor necrosis factor (TNF)-α and suppressors of cytokine-signaling 3 (SOCS3) levels, which subsequently inhabit insulin signaling through the phosphorylation of IRS-1^Ser307^, a key component of insulin signaling transduction (39). Meanwhile, Silence of insulin receptor substrate (IRS)-1 increases cell death in retinal Müller cells (40, 41). In turn, the mutation of the serine 307 site on IRS-1 could block the inhibitory actions of TNF-α and SOCS3 in insulin signaling, and thereby prevent apoptosis of rat retinal Müller cells (rMC-1) (42). These studies suggests that drug targeted insulin signaling (such as certain IRS-1 sites) could be effective in protecting against diabetic damage to retina.

### 4.2 Glaucoma

Glaucoma represents a group of neurodegenerative diseases characterized by progressive retinal ganglion cells dysfunction and death. Although intraocular pressure (IOP) is the only modifiable risk factor for glaucoma patients, hypotensive therapies are not sufficient to prevent optic nerve degeneration and visual loss. Obviously, other pathological mechanisms are involved. Despite inconsistent evidence of the association between glaucoma and diabetes (43–45), people with diabetes might be at an increased risk of developing glaucoma after adjusting for some confounding values (46–48). The intrinsic mechanism of this connection could be attributed to IR, a comorbidity of a constellation of metabolic abnormalities (49). IR in the retina involves damage to neurons, blood vessels, and glia [(50)]. A Mendelian randomization study showed that a genetic predisposition to T2DM was associated with an increased risk of primary open angle glaucoma (POAG) in the European population [(51)]. A nationwide population-based longitudinal cohort study found that diabetes status is a predictor of glaucoma development in postmenopausal women. In particular, patients treated with insulin were associated with higher risk of glaucoma (52). Potential reasons for this phenomenon could be that the use of insulin may be a marker for diabetes severity indicating insulin resistance and high glycemic burden (52). The interplay between diabetes and glaucoma, especially POAG, implies that underlying physiological mechanism of IR in diabetes may contribute to this correlation. Possible mechanisms were also postulated in previous studies, including increased IOP, vascular mechanism, and tissue remodeling of the trabecular meshwork and lamina cribrosa (53, 54). An experimental study showed that IR in the CNS induced by intracerebroventricular injection of S961, a potent and specific blocker of insulin receptor, existed independent of systemic IR and overt diabetes (10). In this study, ocular tissue as an extension of the brain was involved in a series of pathological processes brought about by downregulated insulin signaling, including elevated IOP, morphological changes of the trabecular meshwork, ciliary body dysfunction, and apoptosis in the retina and optic nerve (10). This suggests that IR plays a critical role in the pathogenesis of glaucoma, and improving insulin sensitivity could be a potential therapeutic target.

IR could increase the risk of glaucoma through damage to retinal ganglion cells (RGCs) and axons directly or through glial dysfunction and IOP elevation indirectly. Decreasing insulin sensitivity disrupts the balance of insulin signaling transduction, particularly mediated by the PI3K/Akt pathway which serves as a central component modulating insulin-induced neuronal survival (50). In RGCs, activated Akt prevents the transcriptional activity of p53 and decreases the expression of pro-apoptotic proteins, such as Bad, caspase 9, and glycogen synthase kinase 3 beta (GSK3β) (55). And dendritic retraction of RGCs is one of the earliest pathological changes in glaucoma (56). Agostinone et al. demonstrated that insulin signaling promotes dendrite and synapse regeneration after axonal injury through insulin-dependent mTOR1/2 activation (57). Other potential biological mechanisms explaining IR-induced RGCs impairment in glaucoma include mitochondrial dysfunction, Tau hyperphosphorylation, and amyloid disposition (50).

In addition to RGCs themselves, extracellular environment is also crucial for RGCs survival and axonal transduction, including vascular and glial components. In IR conditions, the imbalance between NO and endothelin-1 via PI3K and MAPK-dependent signaling may underlie the vascular endothelial disturbances in glaucoma (58), corresponding to the vascular theory in the development of glaucoma resulting from decreasing perfusion of the optic nerve and intraneural ischemia. Insulin can affect glial activation, participating in neuroinflammation, which is an inextricable pathological process with IR in neurodegenerative diseases (59). Müller cells, astrocytes, and microglial cells are three major types of glia in the retina that maintain retinal homeostasis by taking part in neuroinflammation and regulating neurotrophic molecules (60). Under pathological conditions, reactive activation of Müller cells is involved in inflammation and cell survival processes due to their intrinsic role in counteracting stress damage (61). In addition, Müller cells could regulate the process of oxidative stress through the expression of glutathione (GSH) and glutamate transporters (62, 63). Meanwhile, as mentioned above, insulin signaling pathways (particularly through the PI3K/Akt pathway) in Müller cells are crucial in the defense of oxidative and inflammatory damage (41, 42). Astrocytes, positioned between the vasculature and neurons, form a cellular network with neurons and other cell types to integrate insulin signaling responses in retina. Decreasing insulin signaling has been shown to impact glycogen synthesis (decreasing expression of the insulin-responsive glucose transporter, GLUT-4) and metabolite redistribution (decreasing gap junctions composed of connexin 43 (Cx43)) in astrocytes (64–66). Like other neuronal cells in the central nervous system, RGCs require high energetic and metabolic support. It is conceivable that any pathological changes in the extracellular milieu could influence the survival and functions of RGCs, which would become more susceptible to mechanical (IOP elevation) and chemical (inflammatory factors) stress. Collectively, improving IR or insulin signaling activation could be a promising target for glaucoma treatment.

### 4.3 Age related macular degeneration

AMD is a leading cause of irreversible vision loss in the elderly population in developed countries (67). Aging worldwide leads to an exponential growth of individuals affected by AMD, and the projected number of this population is 288 million in 2024 (68). AMD can be classified into two forms: dry (also known as nonexudative or atrophic) and wet (also known as exudative and or neovascular). Early AMD is usually asymptomatic, while severe vision loss occurs rapidly in late AMD, which could be further categorized into neovascular AMD and geographic atrophy. The pathogenesis of AMD is complicated with multiple risk factors, including age, genetic variation, systemic diseases, diet, obesity and smoking (69). At present, anti-vascular endothelial growth factor (VEGF) is the first-line treatment for wet AMD, but there is no effective treatment for early AMD or delaying its progression.

According to the International Diabetes Federation (IDF) criteria, MetS represents a constellation of metabolic abnormalities involving centrally distributed obesity, decreased high-density lipoprotein cholesterol, elevated triglycerides, elevated blood pressure, and hyperglycemia. Among these components of MetS, IR is a core feature (70). MetS has long been postulated to be associated with AMD. A previous study provided evidence that a wild-type mouse model of MetS fed with a “fast food” diet showed retinal ultrastructural changes relevant to AMD, including basal laminar deposits, focal thickening of Bruch’s membrane, and a significant loss of retinal pigment epithelium (RPE) cells (71). This *in vivo* study suggests that a “fast food’’ diet mimicking MetS is sufficient to induce altered retinal morphology, as some features seen in AMD. Furthermore, some epidemiologic evidence from the Blue Mountains Eye Study supported that MetS, obesity, high glucose, and triglycerides were predictors of progression to late AMD with a 10-year follow-up period (72). In addition to MetS, the interplay between AMD and diabetes has attracted increasing attention from various data sources, designs, and ethnic groups (73–77).

Although the potential mechanisms have not been fully elucidated, the increased risk of AMD in individuals with MetS or diabetes is thought to be associated with IR-induced oxidative stress with the accumulation of advanced glycation end products (AGEs) in multiple tissues, including photoreceptors, RPE, Bruch’s membrane, and choroidal circulation (74). Second, IR associated metabolic abnormalities, like hyperglycemia and dyslipidemia, disrupt the homeostasis of the retina by inducing inflammatory responses (78). The accumulation of AGEs and inflammatory activations would lead to RPE/photoreceptors dysfunction and even cellular death, outer retinal hypoxia, and a higher likelihood of developing drusen and abnormal deposits (74, 79, 80). Third, AGEs accumulation may upregulate VEGF expression in RPE cells, which plays an important role in the neovascularization of both exudative AMD and diabetic retinopathy (75, 81). Recently, relevant studies demonstrated a dose-dependent decrease in developing AMD among participants who had taken metformin, the first-line medication used to treat T2DM patients and improve insulin sensitivity (82, 83). These results provide evidence from another perspective regarding the key role of IR in the occurrence and progression of AMD, and improving IR may exert a beneficial impact on the management of AMD patients. Moreover, a biomedical study presented that unlike RPE cells obtained from non-AMD normal control eyes, insulin signaling transduction through phosphorylated ERK 1/2 was impaired in the proliferation of RPE cells from patients with AMD (84). Since insulin is a mitogen for human RPE cells (85), further investigation of insulin related kinase signaling abnormalities in RPE cells from AMD patients may help explore new therapeutic targets.

There is no denying that these data provide new insights into the pathogenesis of AMD, although inconsistent opinions exist regarding the relationships between MetS/T2DM and AMD progression (73, 86–88). As mentioned above, either MetS or T2DM is not a distinct disease entity but a sequence of broader underlying metabolic abnormalities characterized by IR. Herein, we may conclude that inherent IR-related molecular mechanisms in MetS/T2DM patients aggravate the occurrence and progression of AMD. Taken together, further epidemiological and longitudinal studies are needed to investigate the exact relationships between AMD and MetS or T2DM. More biological experiments involving IR associated signal pathways would help elucidate the molecular mechanisms behind these relationships and provide additional strategies for the prevention of AMD.

## 5 Possible mechanisms of IR associated with retinopathies

**Figure 2:**
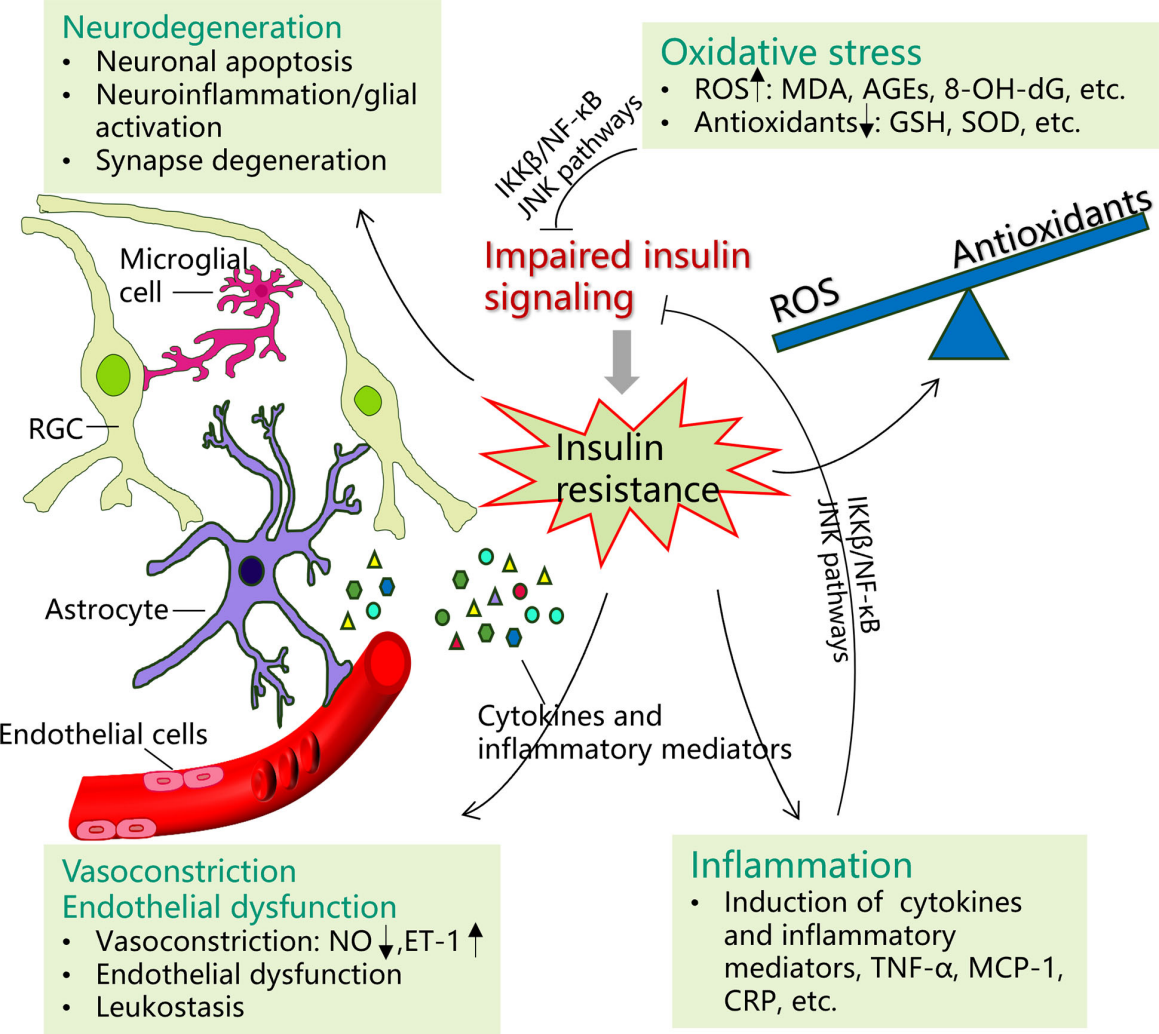
Potential mechanisms of insulin resistance on certain ocular diseases. Possible pathophysiological mechanisms of the interplay between insulin resistance (IR) and certain ocular diseases may include oxidative stress, inflammation, vasoconstriction and endothelial dysfunction, and neurodegeneration. Insulin resistance promotes the production of reactive oxygen species (ROS) (malondialdehyde (MDA), advanced glycation end products (AGEs), 8‐hydroxy‐2′‐deoxyguanosine (8‐OH‐dG), etc.), while the production of antioxidants is decreased, such as glutathione (GSH) and superoxide oxidase (SOD). The imbalance between the ROS and antioxidants would cause oxidative stress, which in turn induce IR by impairing insulin signal transduction. Many inflammatory factors and cytokines, particularly tumor necrosis factor-α (TNF-α), monocyte chemotactic protein-1 (MCP-1), and C-reactive protein (CRP), are upregulated in IR. In turn, both inflammatory agents and oxidative stress can induce IR through activating IKK-β/NF-κB and JNK pathways. Under IR, increasing neuronal apoptosis, glial reactive activation (upregulated neural inflammation), and synapse degeneration are involved. IR additionally contributes to vascular dysfunction by causing nitric oxide/endothelin-1 imbalance (vasoconstriction) and endothelial cell dysfunction.

As insulin signaling transduction is complicated, involving many enzymes and proteins, any disturbances in the transduction pathways would cause IR. While the exact physiological mechanisms of the interplay between IR and associated retinopathies have not been completely ascertained, potential mechanisms of oxidative stress, subclinical inflammation, vascular mechanisms, and neural impairments may be involved (**Figure 2**). In this review, we would explore these mechanisms in detail as below. In addition, it should be noted that some other mechanisms have also been postulated previously, including mitochondrial dysfunction, endoplasmic reticulum stress, etc (6).

### 5.1 Oxidative stress

Although retinal abnormalities in IR patients could be explained by chronic dyslipidemia, hyperinsulinemia and adipose inflammation, the core mechanism of these factors could be attributed to mitochondrial dysfunction and oxidative stress (89). Indeed, there is emerging evidence that retinopathy in IR models is initiated and propagated by multiple metabolic toxicities associated with excess production of reactive oxygen species (ROS) (90). Fernandes and colleagues observed increased oxidative stress and peroxynitrite-mediated protein oxidation in retinas from diabetic GK rats, a model characterized by mild hyperglycemia, insulin resistance, normolipidemia, and hyperinsulinemia (91). An increase in markers of oxidative stress, including gp91phox, MDA and 8OH-dG, was observed in the inner retina of high-fat diet (HFD) mice, indicating increased oxidative DNA and lipid peroxidation injury were present in obese and insulin-resistant animals compared to chow diet (CD) fed controls (92). Generally, oxidative stress is defined as an imbalance between the production of reactive oxygen species (ROS) and endogenous antioxidants, which includes enzymic antioxidants such as superoxide oxidase (SOD), catalase (CAT) and glutathione peroxidase, and non-enzymic antioxidants such as GSH (93–95). Accordingly, when the redox balance is upset, the oxidative damage would occur in which the level of ROS exceeds the capacity of antioxidative defense system to neutralize them. Overproduction of ROS results in oxidative damage to cell structure and subsequently modifying cell function, including lipids loss and increased membrane permeability, modifying nucleic acids leading to mutations and apoptosis, as well as oxidatively modified proteins (96). Collectively, hyperglycemia, oxidative stress, and redox homeostasis changes are fundamental events in the pathogenesis of IR associated retinopathies, contributing to the production of pro-inflammatory cytokines, abnormalities of multiple retinal cells, and extracellular matrix remodeling (97).

Under IR circumstances, disturbances of oxidative and antioxidative products have been reported in the tissues of liver, skeletal muscle, kidney and brain (22, 98–100). Additionally, the retina is particularly sensitive to oxidative stress, considering the high oxygen demand and consumption and its high concentration of polyunsaturated fatty acids (PUFA) in the outer segments of photoreceptor (101). Although PUFA are thought to protect against oxidative and inflammatory damage, they may also serve as a substrate for free radicals under special conditions as they provide an available source of electrons (94). Therefore, oxidative stress under IR conditions with overproduction of ROS plays a prominent role in retinopathies.

### 5.2 Chronic low-grade inflammation

It is recognized that subclinical chronic inflammation and activation of the immune system are involved in the pathogenesis of IR-related metabolic disorders and even seem to be independent risk factors (102). Pro-inflammatory cytokines (interleukin (IL)-1β, IL-6, and TNF-α), chemokines, and acute-phase proteins such as C-reactive protein (CRP) are elevated in IR (103, 104). These pro-inflammatory makers promote IR by interfering with the c-JUN N-terminal kinase (JNK), nuclear factor-kappa B (NF-κB), and the NADPH oxidative pathways (105), which could activate serine phosphorylation of IRS-1 and further block insulin signaling proteins (such as PI3K-Akt and AMPK) (106). Meanwhile, inflammatory reaction and oxidative stress are two interrelated processes (79). In this process, the activation of oxidative stress may regulate inflammatory genes and lead to inflammation, which would in turn aggregate oxidative stress. In addition, it is reported that SOCS proteins induced by inflammation cytokines can trigger IR and inhibit pro-survival insulin signaling pathways in retina (20), supporting that SOCS-mediated IRS degradation inhabits insulin signaling transduction via insulin receptor. Accordingly, these studies provide evidence about a link between IR and inflammation.

### 5.3 Endothelial dysfunction and vasoconstriction

Endothelial dysfunction, closely related to IR, is the core of microvascular complications (107). To some extent, IR has a close relationship with systemic metabolic abnormalities, such as hyperglycemia and dyslipidemia, so a link between IR and microvascular dysfunction is not surprising. Insulin has been known to play a direct role in microvascular physiology. In endothelial cells, insulin-mediated Akt activation exerts antiapoptotic effects via phosphorylation of caspase-9 (108). Insulin is a potent vasoactive hormone, which is demonstrated to stimulate the expression and activation of endothelial NO synthase via PI3K pathway (50). Endothelial cell-mediated autoregulation of blood flow is sustained by a balance between NO-dependent vasodilation and the activation of vasoconstrictor mechanisms, including endothelin-1 (ET-1), sympathetic activity, and the renin-angiotensin system (21). In IR conditions, impairments of the PI3K and AMPK-dependent signaling may cause an imbalance between NO and ET-1 and lead to endothelial dysfunction and ischemic injury (109). Moreover, retinal leukostasis, an important contributor to capillary occlusion in ischemic retinopathies, is correlated to endothelial dysfunction and could be enhanced by IR independently (110).

### 5.4 Neurodegenerative impairments

Insulin and insulin-like growth factor have been reported to play a role in the survival of RGCs, which are responsible for transmitting visual information together with their axons from the eye to the brain (5). That is, functional insulin and downstream elements in insulin signaling pathways are required in neurodegenerative diseases. In addition to vascular theory, neurodegenerative impairments have been increasingly recognized among the diseases listed above, no matter whether primary or secondary to primary disorders. With regard to neurodegenerative diseases, the term “neurovascular unit” was put forward and first applied to blood-brain barrier and then to retina, which refers to the functional coupling and interdependency of neurons, glia, and vasculature (111). Based on this concept, retinal neurodegenerative diseases not only include neural-cell apoptosis but also glial activation and neural function impairments. It is suggested that neuroinflammation and microglial activation play a central role in the pathophysiology of neurodegenerative diseases (112). Consistent with that, microglial cells are shown to be the main source of ROS, TNF-α, and glutamate, all of which are neurotoxic when released at a high level after glial activation induced by the stimulus of toll-like receptors (TLRs) and NF-κB (113, 114). As mentioned above, PI3K and Akt are identified as central components mediating downstream insulin-induced neuronal survival, neuroinflammation, and dendritic regeneration (50). In an experimental study of type 2 diabetic rats, insulin receptors and downstream Akt signaling were found to regulate neural cell survival (115). In addition, although the exact mechanism has not yet been elucidated, IR is suggested to be associated with elevated intraocular pressure (IOP) (116, 117), subsequently causing mechanical impairments for RGCs. Therefore, targeting activation of insulin signaling and improve IR could serve as effective neuro-protective therapy.

## 6 Potential treatment target of IR in retinal diseases

In the CNS, restoring insulin signaling has significantly prevented cognitive decline in Alzheimer’s disease (AD) through the administration of intranasal insulin without altering blood insulin and glucose levels (118). IR is shown to be directly correlated with AD pathology, including the production and clearance of Aβ peptide, tau hyperphosphorylation, immune function, and inflammatory markers (119, 120). As listed above, though the occurrence of DR, AMD, and glaucoma is multifactorial with distinct clinical features, IR may be a common risk factor causing retinal impairments through pathophysiological mechanisms involving oxidative stress, chronic inflammation, vascular endothelial dysfunction, and retinal neural loss, etc. Moreover, in addition to above retinal diseases we have reviewed, retinal vein occlusion has also been proposed to be inextricably linked to IR (49). Thus, it is conceivable that the actions of insulin signaling pathway exert an important effect on the viability of retinal cells through the activation of downstream pro-survival kinases; thereby, the restoration of insulin signaling transduction could be an underlying therapeutic target in certain retinal diseases. This point is of vital importance for the early management of patients with retinal diseases, given that current available treatments in retinal diseases could not achieve desired results. For example, either surgical intervention or retinal photocoagulation is largely destructive, both of which only delay disease progression. There is, however, a small percentage of eyes (15–40%) that fail to respond or only partially respond to intravitreal injections of vascular endothelial growth factor antagonists (121).

In fact, IR is not equal to hypoinsulinemia, but a decreased sensitivity of insulin receptors and impaired activation of specific downstream pathways. As stated in previous studies, in insulin-responsive tissues such as the heart, liver, and adipose tissue, IR is an adaptive defense mechanism that, to some extent, could be able to protect the tissues from nutrient-induced damage (122). Insulin-induced metabolic stress is likely to occur with systemic high-dose insulin therapy without nutrient off-loading in refractory patients (123). Therefore, no matter whether it is systemic treatment or topical retinal administration, improving IR not only means intensive exogenous insulin usage, but also improves the sensitivity of insulin receptors and the transduction of certain insulin pathways. Undoubtedly, insulin, a primary anabolic peptide, could play a key role in the regulation of retinal functions. Nevertheless, it should be emphasized that insulin signaling pathways are complex, in which selective activation or specific mutation may cause different phenotypes. Many detailed mechanisms of insulin signaling molecules remain undiscovered currently and deserve further investigation. Another alternative approach to improve IR may be lifestyle modification aimed at losing weight through restricted calorie intake and increased physical activity (71, 124). Future prospective longitudinal studies are also needed to validate whether lifestyle intervention would be effective to reduce the incidence and delay the progression of retinal diseases.

## 7 Conclusion

IR could be defined as reduced insulin sensitivity in target organs, which represents the comorbidity of a sequence of metabolic abnormalities, such as obesity, hyperglycemia, and dyslipidemia. In addition to common target organs (liver, skeletal muscle, etc.), the retina has also been demonstrated to be insulin-sensitive, in which insulin participates in many cellular processes and plays a key role in retinal cell survival. Previous studies have provided evidence that IR is closely associated with several retinal diseases, especially DR, AMD, and glaucoma. Possible explanations for these associations include oxidative stress, chronic low-grade inflammation, endothelial dysfunction and vasoconstriction, and neurodegenerative impairments. Thus, it may be important to monitor the IR conditions in populations and identify those at high IR levels. Subsequently, improving IR may provide new insights for the prevention and treatment of certain retinal diseases. Nevertheless, it should be noted that, on the one hand, the insulin signaling pathways are complex, and on the other hand, current prospective studies regarding the role of improving IR in the management of certain retinal diseases are relatively lacking. Consequently, more clinical and experimental studies are warranted to validate the mutual relationships between IR and certain retinal diseases, which would be beneficial for the management of certain ocular diseases.

## Author contributions

ZZ: Conceptualization, Investigation, Writing – original draft. XY: Supervision, Validation, Writing – review & editing.
